# Supplementary material for: Essential gene knockdowns reveal genetic vulnerabilities and antibiotic sensitivities in Acinetobacter baumannii
Source: mBio. 2023 Dec 21;15(2):e02051-23. doi: 10.1128/mbio.02051-23 (PMC10865783; doi:10.1128/mbio.02051-23)
Supplement: Supplemental Figures — Figures S1-S16. [file mbio.02051-23-s0001.pdf]

S1

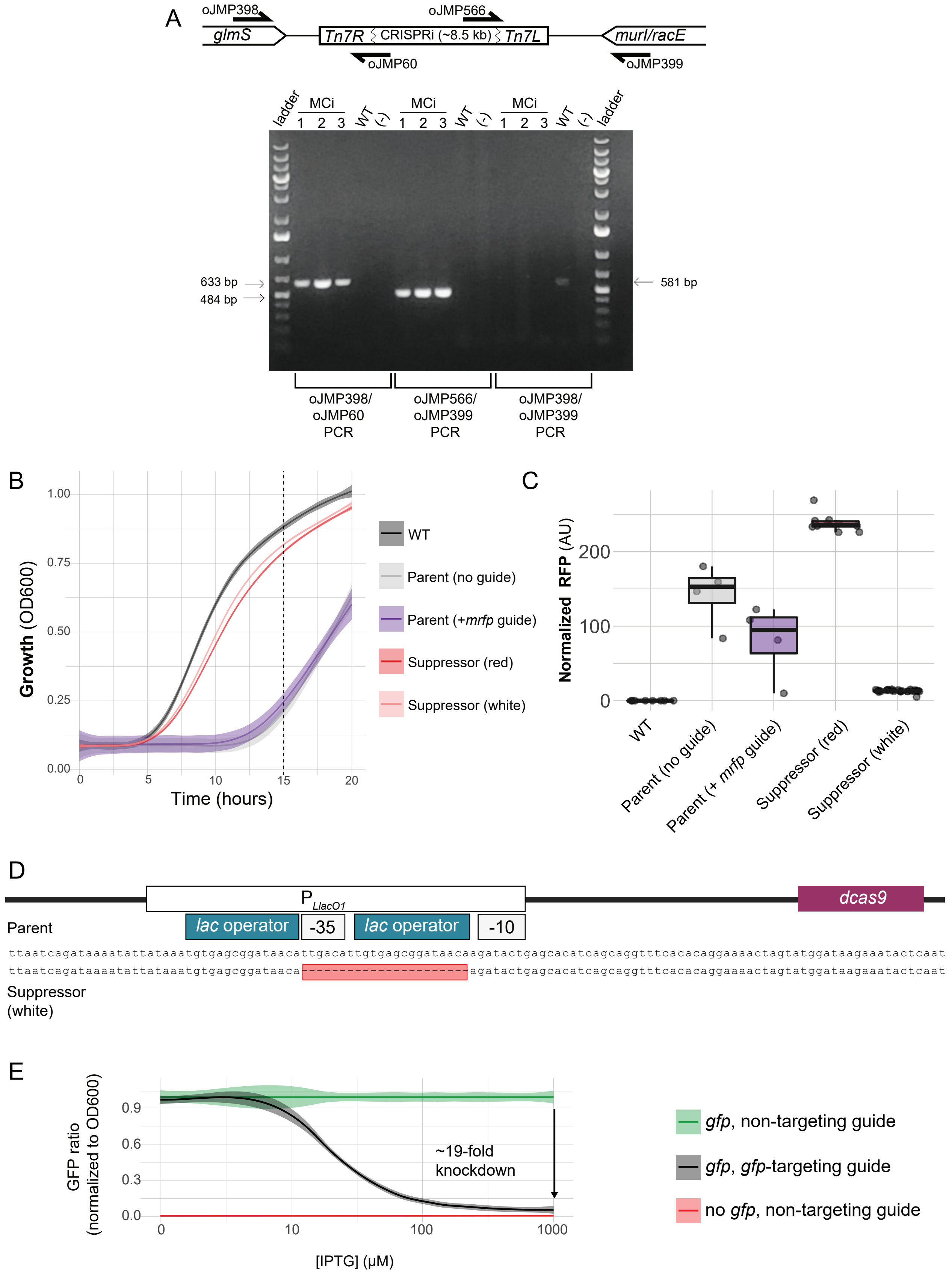

Fig S1 Optimization and characterization of an *A. baumannii* Mobile-CRISPRi system. **(A)** PCR confirmation that Mobile-CRISPRi (MCi) inserted downstream of the *glmS* gene as expected in A. baumannii. **(B)** Expression of dCas9 from the $P_{LlacO\text{-}1}$ promoter is toxic in *A. baumannii* 19606 (Parent (+*mrfp* guide)), but suppressors can readily be obtained that reduce toxicity (Suppressor (red) and Suppressor (white)). White colony suppressors have reduced toxicity of dCas9 expression but are still capable of targeting a chromosomal copy of the gene encoding Red Fluorescent Protein (*mrfp*). **(C)** RFP fluorescence of parent ($P_{LlacO\text{-}1}$-*dcas9*) and suppressor colonies. Each point is a distinct, single colony. Parent strains likely show intermediate levels of RFP due to the toxic effects of dCas9 expression from $P_{LlacO\text{-}1}$. **(D)** White colony suppressors contain a mutation in PLlacO-1 that likely reduces its activity; this mutation likely arose from collapse of identical *lac* operator sequences. **(E)** Knockdown quantification of a CRISPRi system that expresses dCas9 from the white suppressor promoter at varying concentrations of inducer (IPTG).

S2

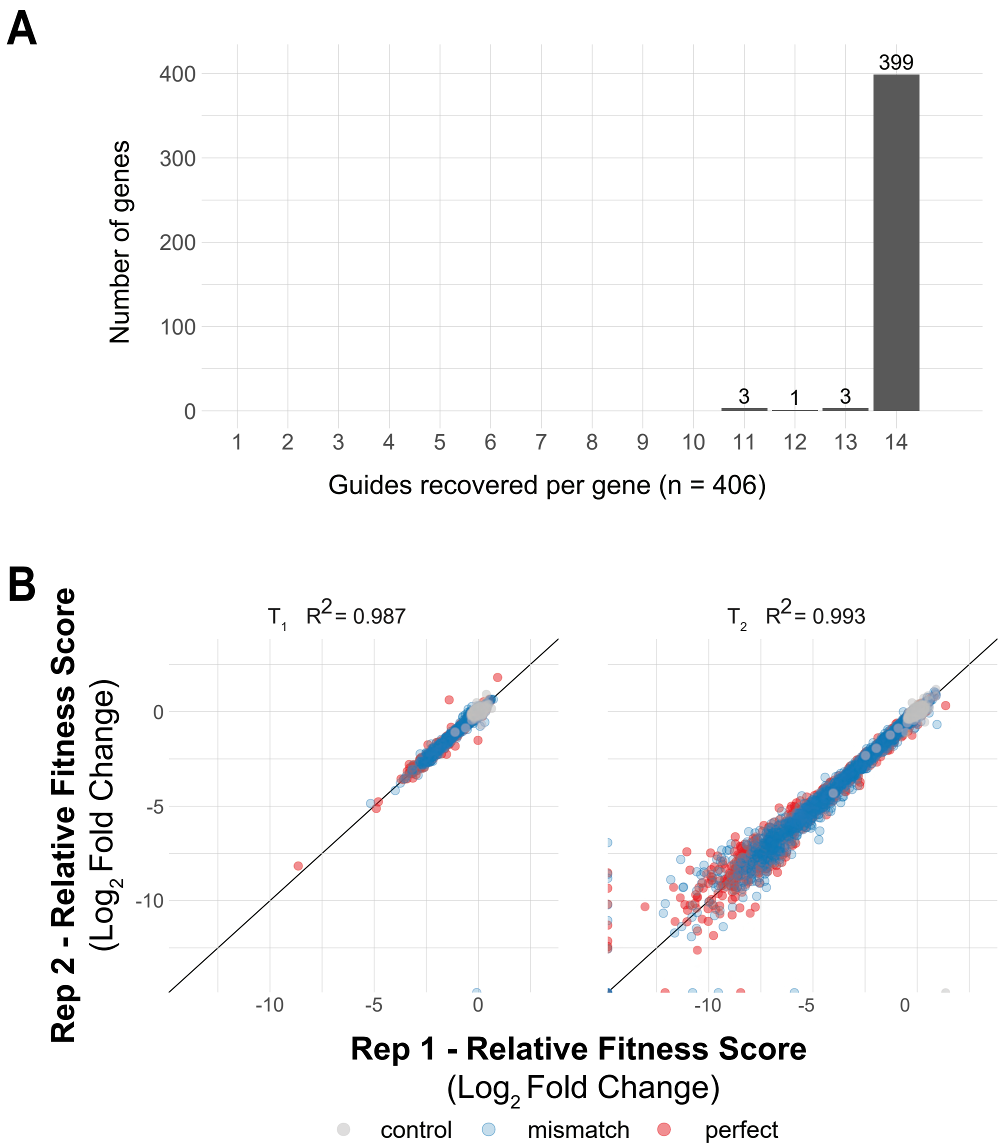

Fig S2 Quality control for the *A. baumannii* essential gene CRISPRi library. **(A)** Histogram of guides/gene in the library. The vast majority of genes had 14 guides and the smallest number of guides/gene was 11. Libraries shown here were grown with IPTG to two separate time points (see Methods) and sgRNA spacer depletion was quantified at each time point. Of the 7 genes with less than 14 recovered guides, 4 genes had only 13 (N=3) or 12 (N=1) guides in our initial library design. The other 3 genes likely lost a targeting guide during the library cloning or mating procedures. **(B)** Biological replicates of CRISPRi library experiments showed excellent reproducibility.

S3

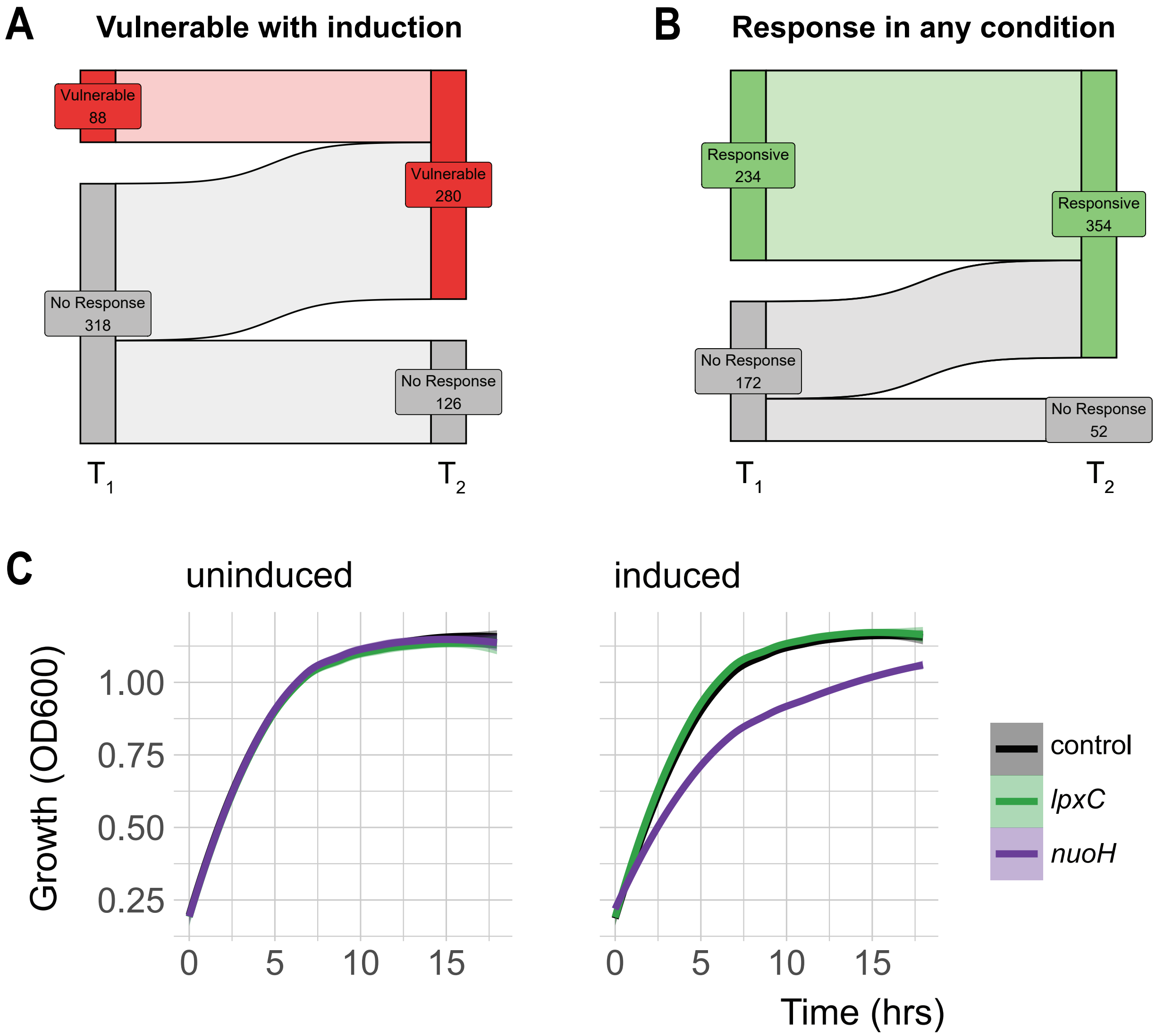

Fig S3 Gene level depletion from the CRISPRi library under different growth conditions. **(A)** Sankey plot of gene depletion at two time points. Genes are considered vulnerable to knockdown if they were depleted by 2-fold with a Stouffer's *p* value of 0.05 at the time point indicated relative to T0. **(B)** Sankey plot of gene depletion across any of the conditions assayed in our experiments (IPTG alone, IMI, MER, COL, RIF). Genes are considered responsive if they were depleted by 2-fold with a Stouffer's *p* value of 0.05 at the time point indicated relative to T0. **(C)** Growth, measured by OD600, over 18 hours for a non-targeting control strain, *lpxC* knockdown strain, and *nuoH* knockdown strain in LB (uninduced, left) or LB with inducer (1 mM IPTG, right).

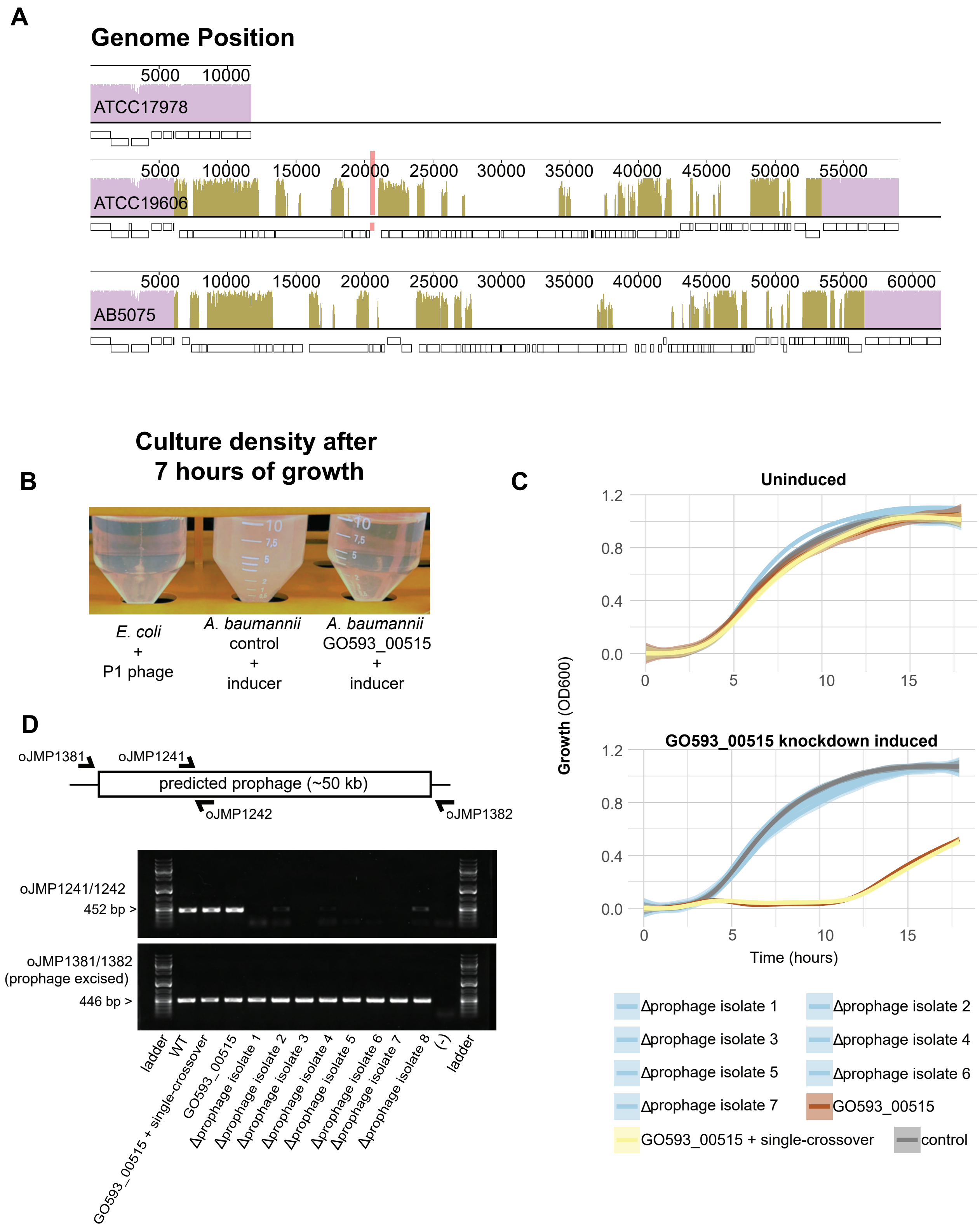

Fig S4 The GO593_00515 gene is conditionally essential. **(A)** Mauve alignment (see Table S4) of *A. baumannii* 19606 genomic locus containing GO593_00515 (pink) and surrounding prophage (yellow) to 17978 and AB5075. Regions of same color represent alignment between genomes. **(B)** Cultures seven hours after addition of P1 phage lysate to *E. coli* MG1655 (left) and addition of inducer (1 mM IPTG) to *A. baumannii* non-targeting control strain (middle) or GO593_00515 knockdown strain (right). **(C)** Growth, measured by OD600, over 18 hours for a non-targeting control strain, GO593_00515 knockdown strain, GO593_00515 knockdown strain with single-crossover of prophage deletion plasmid (intermediate in construction), and multiple strains of GO593_00515 knockdown with deleted prophage in LB (uninduced, top) or LB with inducer (1 mM IPTG, bottom). **(D)** Agarose gel electrophoresis of PCR products to confirm prophage deletion using specified primer sets. GeneRuler 1 kb Plus ladder or template genomic DNA are denoted below each lane. PCR products with oJMP1381/1382 for strains with prophage still present suggests prophage excision happens at some frequency in WT.

S5

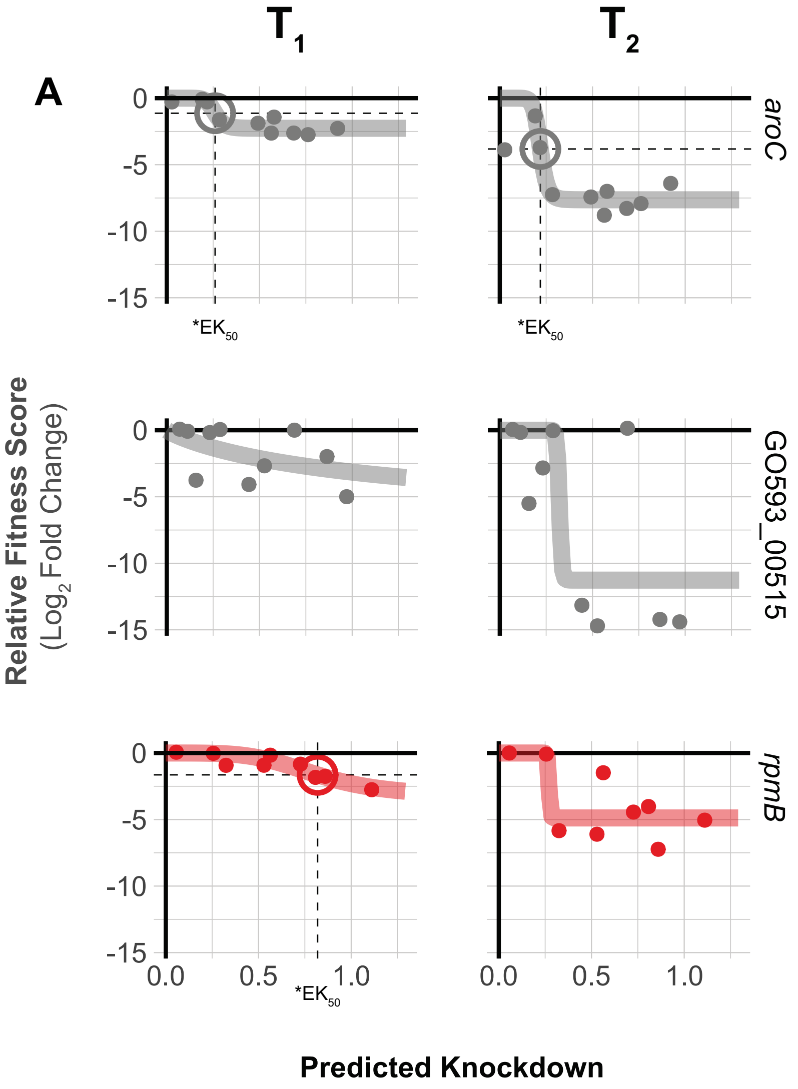

Fig S5 Examples of 4-parameter knockdown-response curves in T_1 and T_2 from genes highlighted in the text. Effective knockdown 50 (EK_{50}) parameters are displayed as crosshairs where the parameter fit resulted in a *p* value ≤ 0.05. Points are individual sgRNAs.

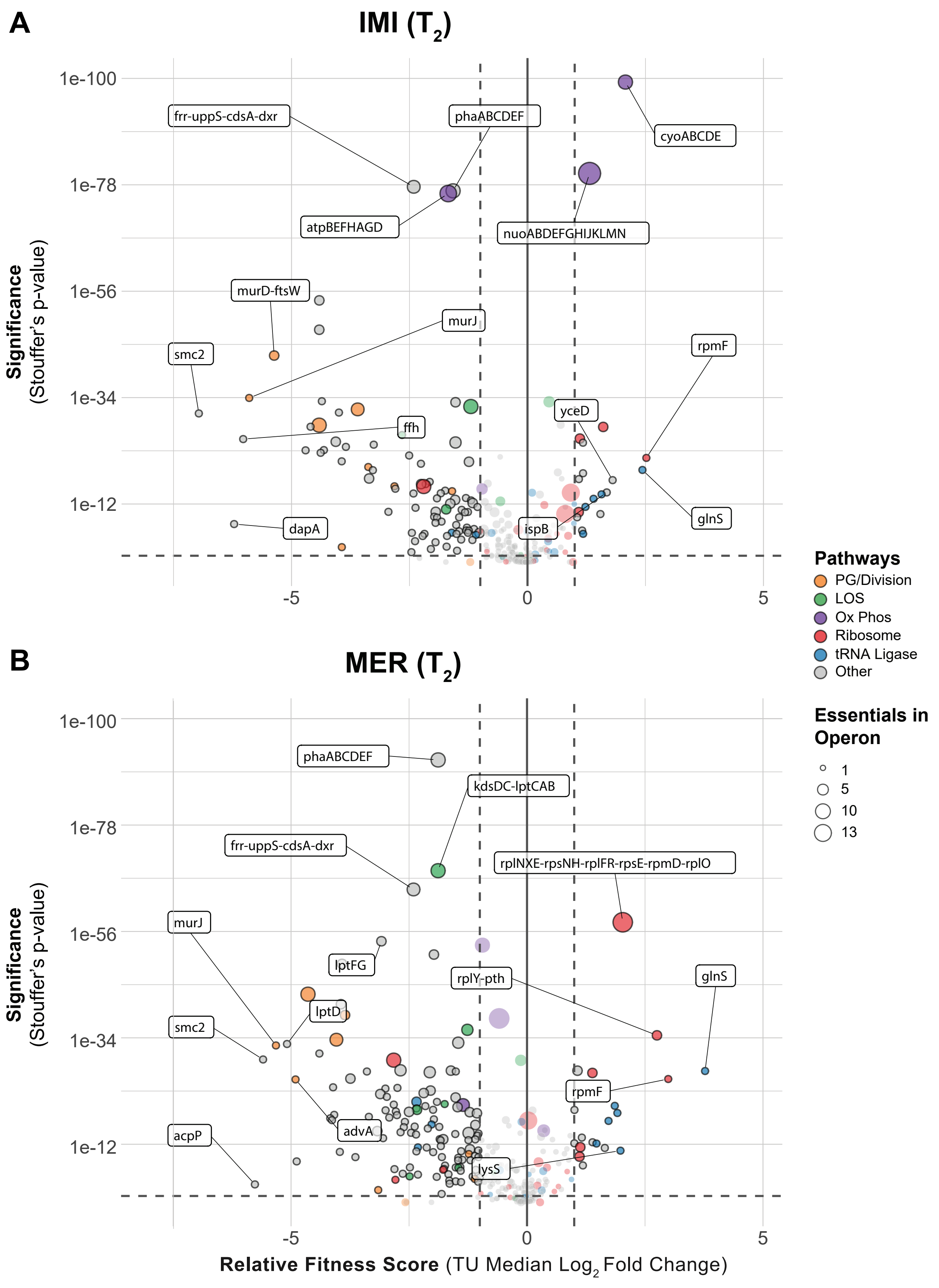

Fig S6 Essential gene knockdown phenotypes in imipenem (IMI) **(A)** and meropenem (MER) **(B)**. Depletion of sgRNA spacers from the CRISPRi library (relative fitness score) during growth in IPTG at the level of transcription units (TUs). Dashed lines indicate a two-fold loss in relative fitness score and a *p* value of ≤ 0.05. Stouffer's *p* values were calculated at the TU level by aggregating false discovery rates (FDRs) of individual sgRNAs targeting the TU. TUs related to pathways discussed in the text are colored as described in the legend and the number of essential genes in the TU is indicated by point size.

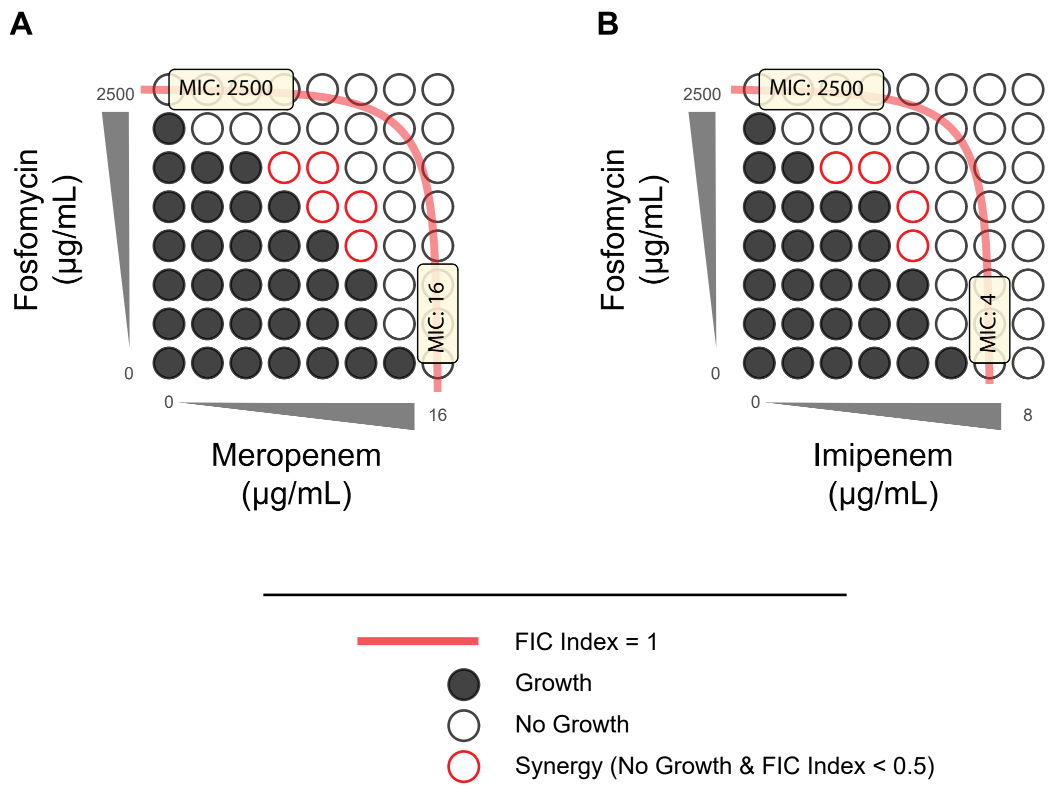

Fig S7 Carbapenems are synergistic with fosfomycin in *A. baumannii*. 2-fold serial dilutions of drugs from minimum inhibitory concentrations (MICs) represented by gray wedges. Wells with red borders show synergy (*i.e.*, no growth & FIC index <0.5).

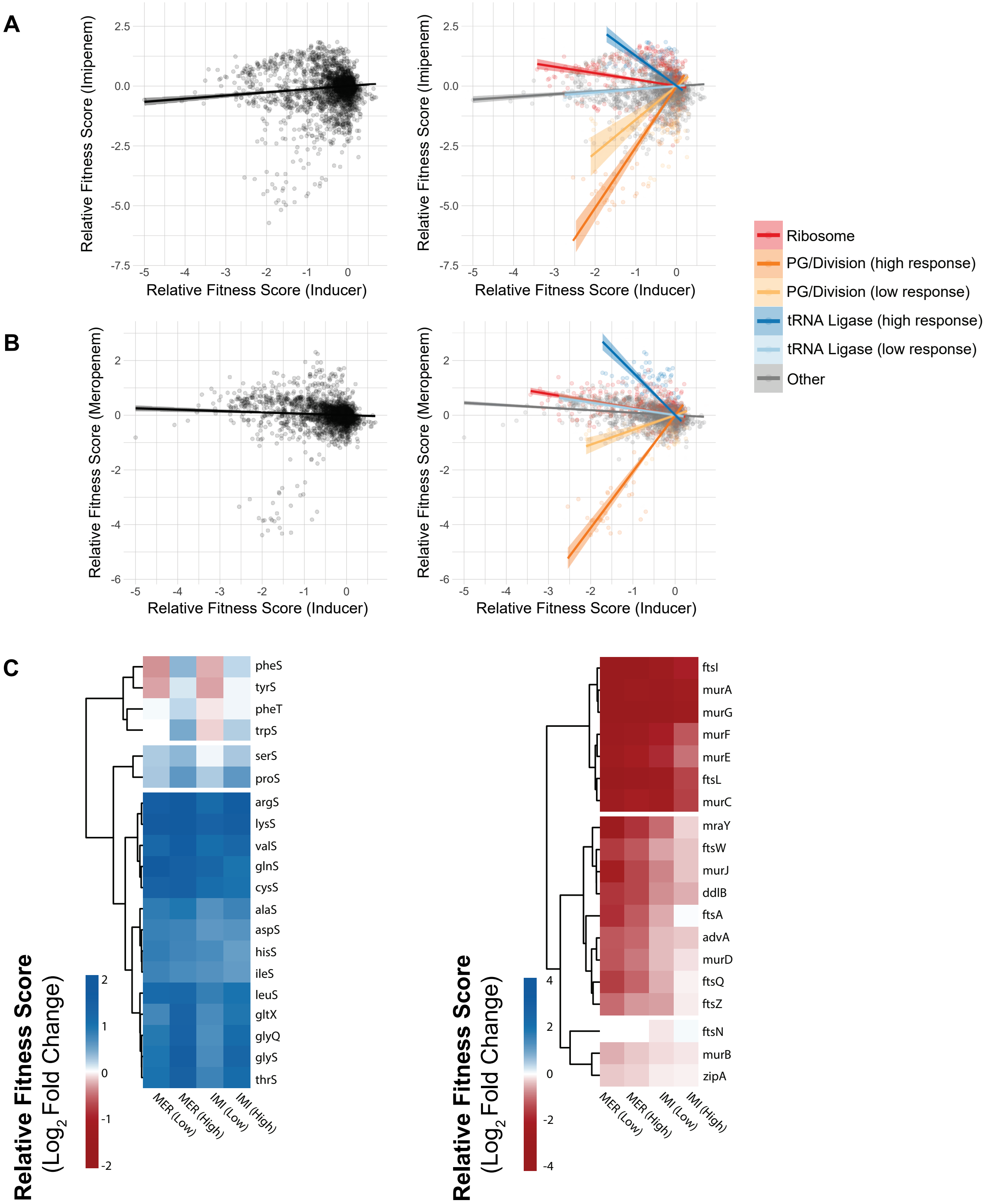

Fig S8 Essential gene knockdown interactions with carbapenems. **(A-B)** Scatterplot of mismatch guide relative fitness score ($\log_2$ fold change) in inducer only compared to relative fitness score in imipenem at T_1. Lines represent linear model fits with 95% confidence interval. Guides for genes in PG/division or tRNA ligase pathways are divided into groups using hierarchical clustering based on response to imipenem. Left-hand figures in grayscale indicate trend for all guides; right-hand figures indicate trends for guides for specific pathways. High-response indicates most responsive cluster (greatest absolute $\log_2$ fold changes), low-response indicates other clusters (k=3) in C. **(C)** Hierarchical clustering of tRNA synthetase and PG/division gene knockdowns in response to imipenem (IMI) and meropenem (MER).

S9

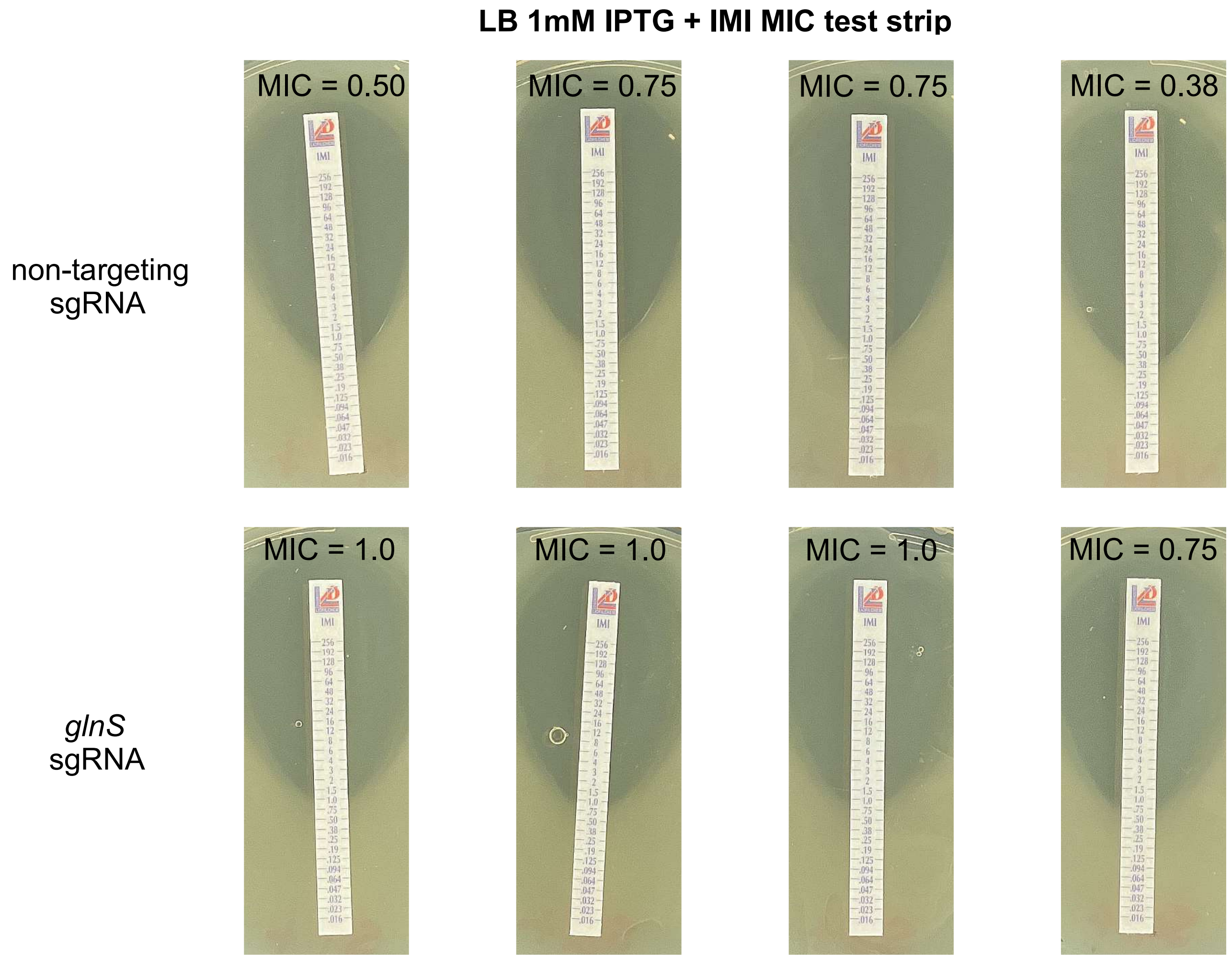

Fig S9 *glnS* knockdown causes a subtle change in IMI MIC. Four independently constructed non-targeting (NT) or *glnS* knockdown strains were plated as a lawn on LB + 1mM IPTG and grown in the presence of an IMI MIC test strip. MIC values were read as the concentration line above the intersection between confluent growth and the test strip. The average MICs for NT and *glnS* were 0.6 and 0.9 ng/µL, respectively (p = 0.02, 2 tailed t-test with equal variance).

S10

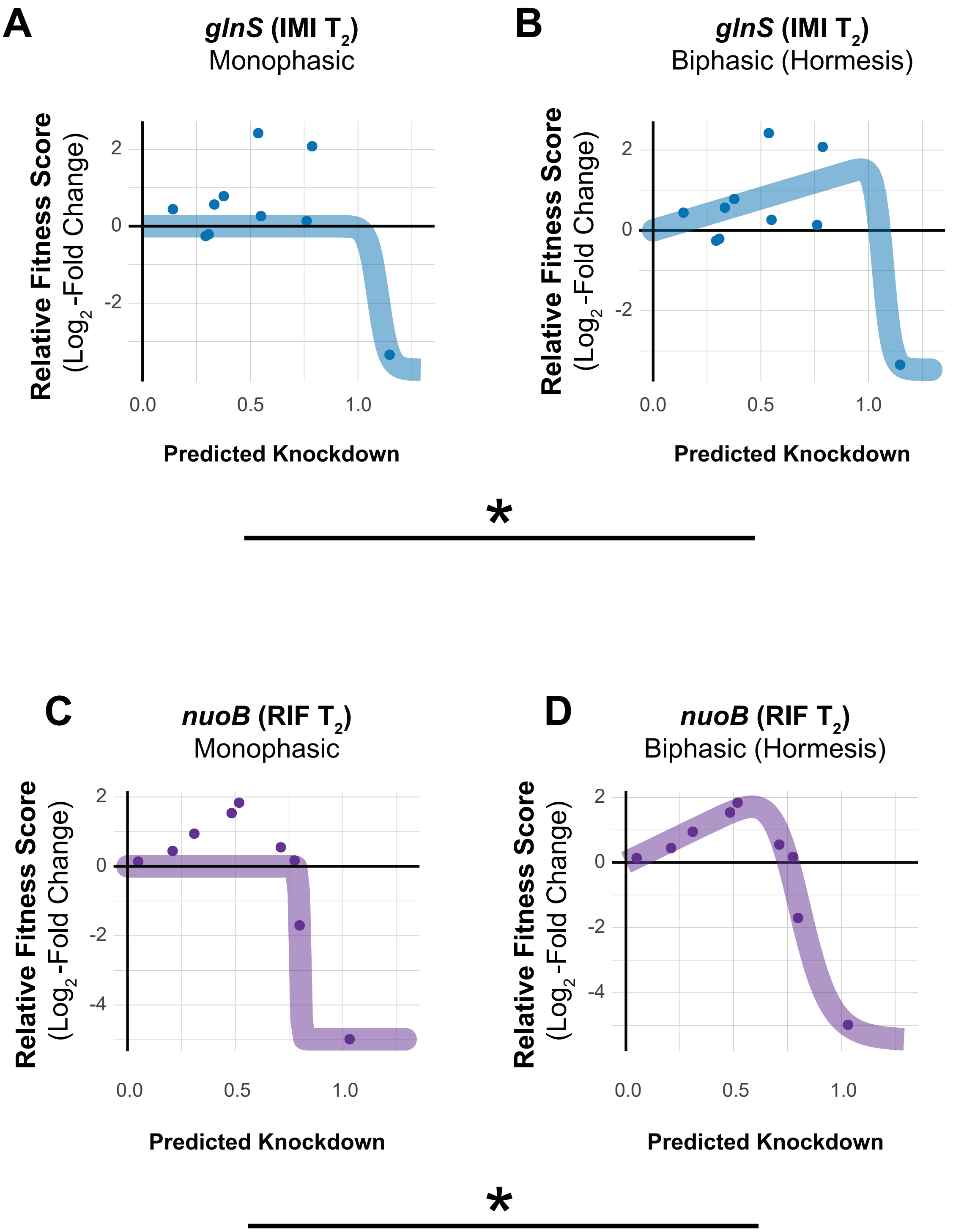

Fig S10 Dose-response curves modeling the fits for *glnS* in imipenem (IMI) **(A-B)** and *nuoB* in rifampicin (RIF) **(C-D)**. Asterisks indicate improvement of the empirical fit from 4-paremeter to 5-parameter, such that the likelihood-ratio test p value ≤ 0.05.

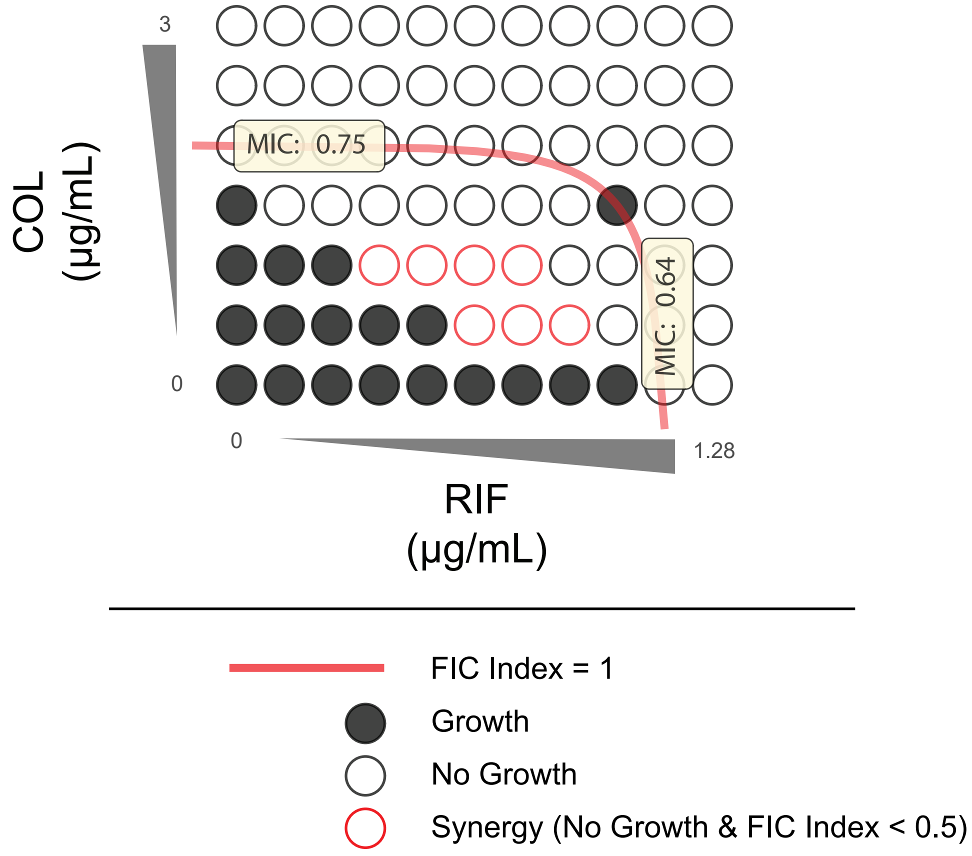

Fig S11 COL and RIF are synergistic under our screening conditions in *A. baumannii*. 2-fold serial dilutions of drugs from minimum inhibitory concentrations (MICs) represented by gray wedges. Wells with red borders show synergy (*i.e.*, no growth & FIC index <0.5).

S12

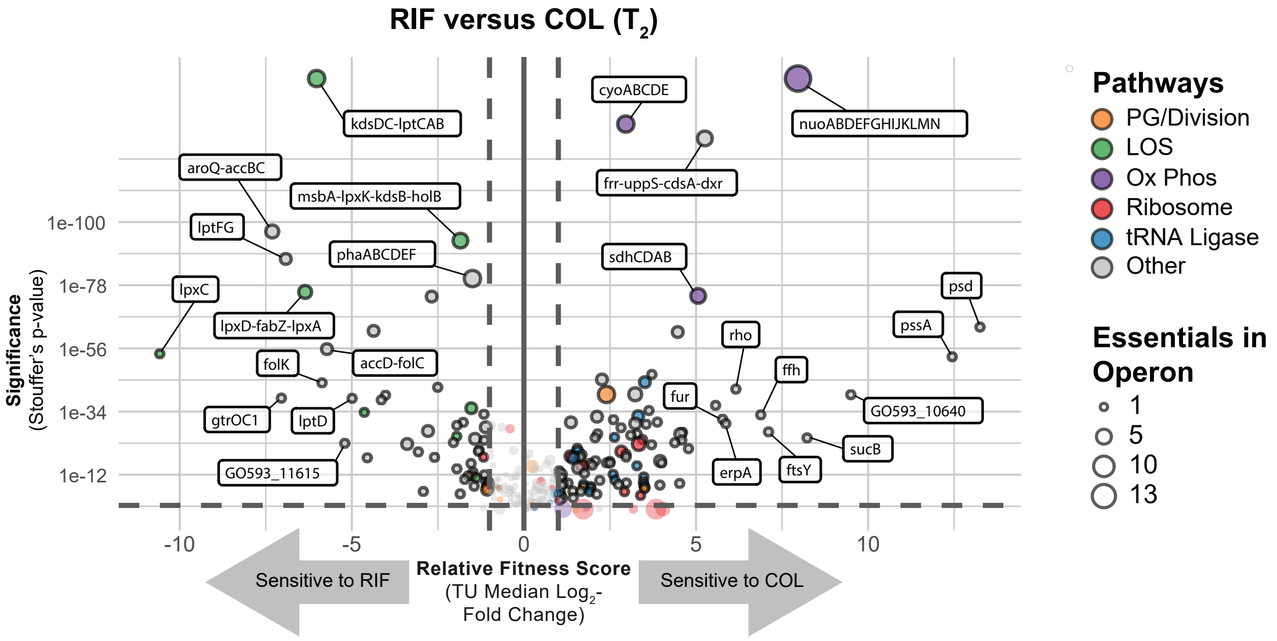

Fig S12 Essential gene knockdown phenotypes in rifampicin (RIF) versus colistin (COL). Depletion of sgRNAs targeting transcription units (TUs) from the CRISPRi library during growth in inducer and RIF or COL at T_2. Vertical dashed lines indicate a two-fold loss in fitness score relative to non-targeting sgRNAs and horizontal dashed lines indicate a Stouffer's *p* value of ≤ 0.05. Stouffer's *p* values were calculated at the TU level by combining the false discovery rates (FDRs) of all individual sgRNAs targeting the TU. TUs related to pathways discussed in the text are colored according to the figure legend and the number of essential genes in a TU is indicated by point size.

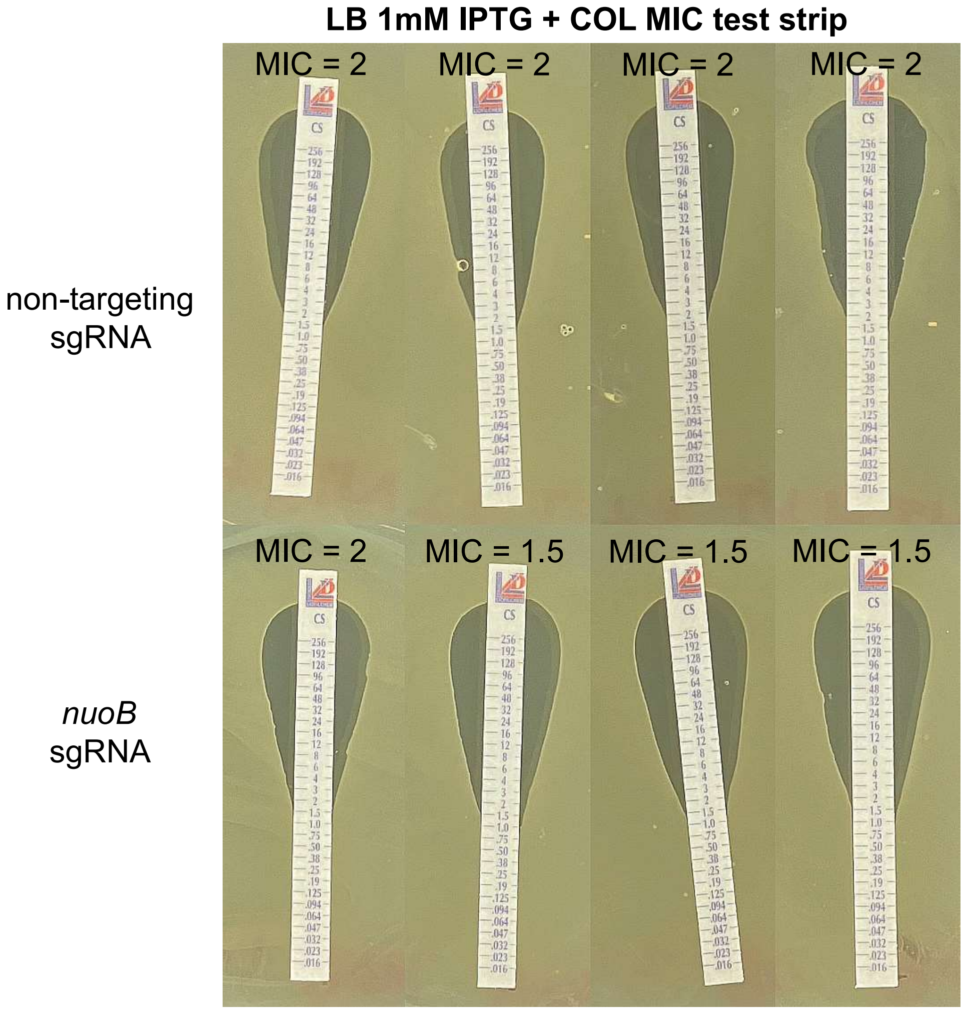

Fig S13 *nuoB* knockdown causes a subtle change in COL MIC. Four independently constructed non-targeting (NT) or *nuoB* knockdown strains were plated as a lawn on LB + 1mM IPTG and grown in the presence of a COL MIC test strip. MIC values were read as the concentration line above the intersection between confluent growth and the test strip. The average MICs for NT and *nuoB* were 2 and 1.6 ng/μL, respectively (p = 0.02, 2 tailed t-test with equal variance).

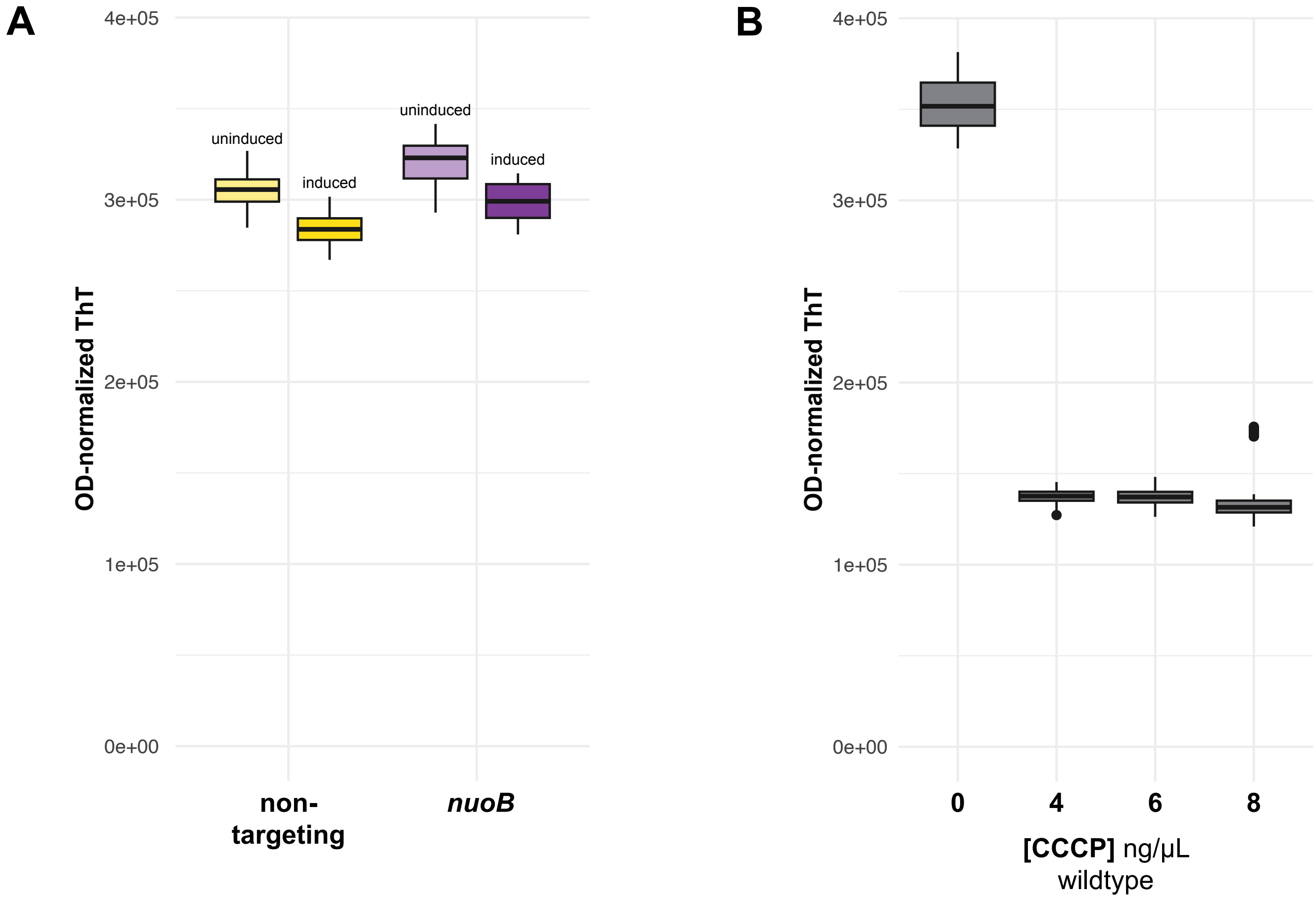

Fig S14 ThT normalized to OD_{600} as a measurement of membrane potential (Δψ) in non-targeting and *nuoB* knockdown strains **(A)** and wildtype **(B)**. Non-targeting and *nuoB* knockdowns measurements are taken with and without inducer **(A)**. Wildtype was treated with CCCP at 0, 4, 6, and 8 ng/µL **(B)**.

S15

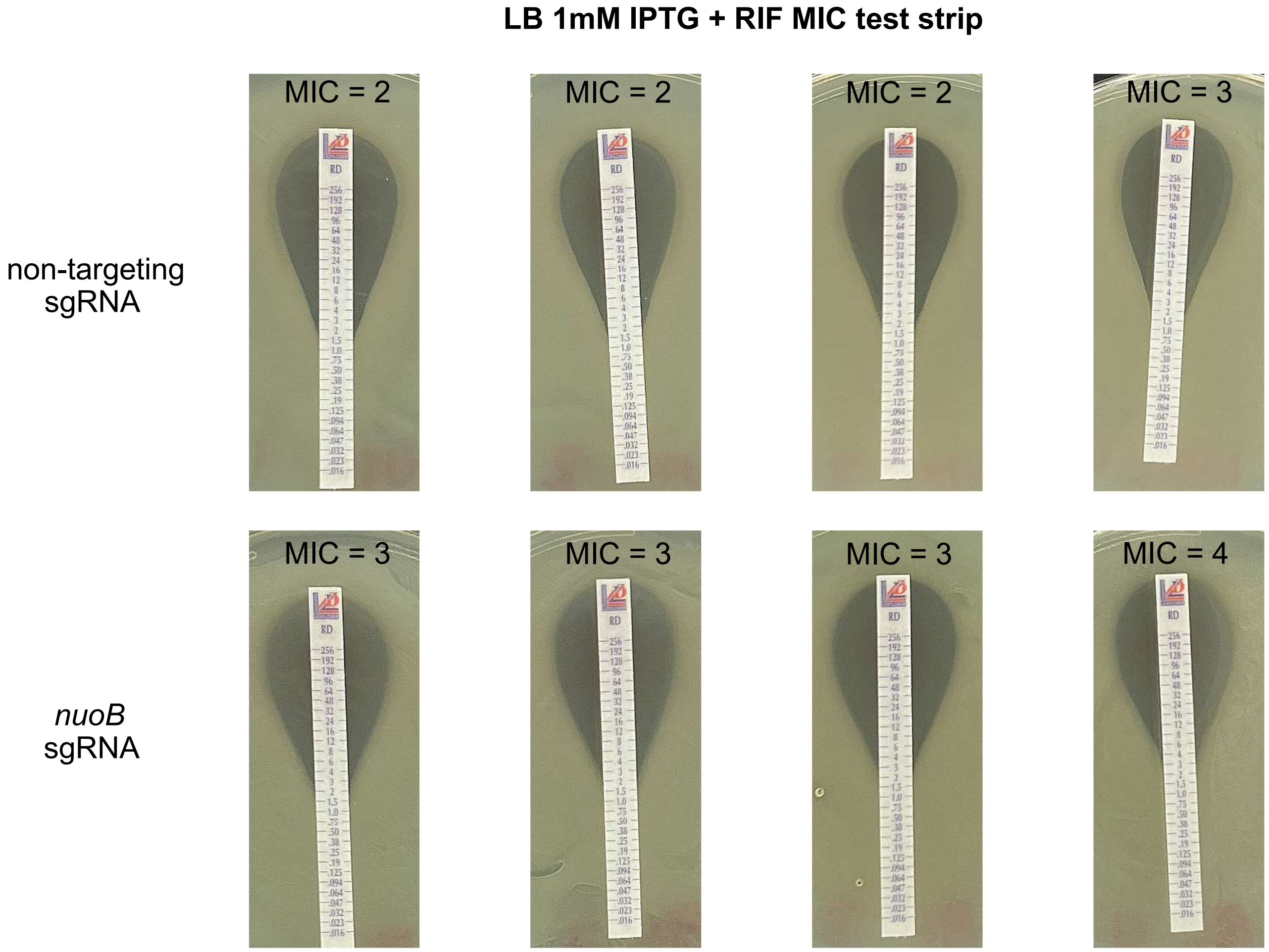

Fig S15 *nuoB* knockdown causes a subtle change in RIF MIC. Four independently constructed non-targeting (NT) or *nuoB* knockdown strains were plated as a lawn on LB + 1mM IPTG and grown in the presence of a RIF MIC test strip. MIC values were read as the concentration line above the intersection between confluent growth and the test strip. The average MICs for NT and *nuoB* were 2.25 and 3.25 ng/μL, respectively (*p* value= 0.03, 2 tailed t-test with equal variance).

A

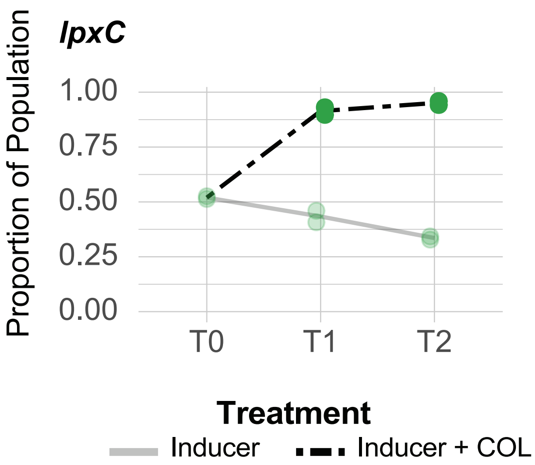

B

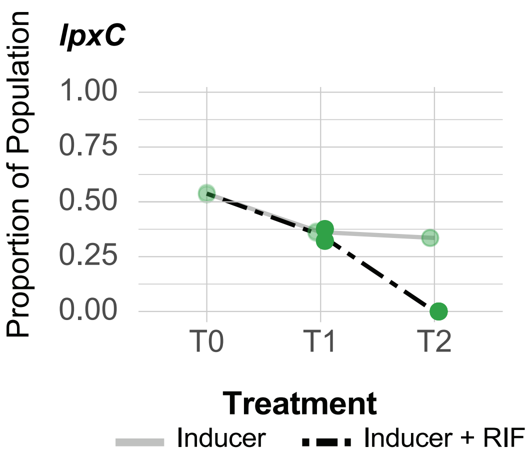

Fig S16 COL/RIF interaction and physiological characterization of NDH-1 knockdown. **(A-B)** CoMBaT-seq experiments competing *nuoB* knockdown with a non-targeting strain in the presence or absence of COL (A) or RIF (B); as the proportion of the non-targeting strain increases, the *nuoB* knockdown decreases and vice versa. Points are data from individual experiments (N=2).
